# Postannealing-Driven Optimization of Humidity Response in Densely and Loosely Grafted Polymer Films

**DOI:** 10.3390/gels12060515

**Published:** 2026-06-10

**Authors:** Katerina Lazarova, Silvia Bozhilova, Martina Docheva, Ketrin Pavlova, Gergana Alexieva, Darinka Christova, Tsvetanka Babeva

**Affiliations:** 1 Institute of Optical Materials and Technologies “Acad. J. Malinowski”, Bulgarian Academy of Sciences, Akad. G. Bonchev Str., Bl. 109, 1113 Sofia, Bulgaria; mdochev@iomt.bas.bg (M.D.); kpavlova@iomt.bas.bg (K.P.); babeva@iomt.bas.bg (T.B.); 2 National Centre of Excellence Mechatronics and Clean Technologies, 8 Kliment Ohridski Blvd, 1756 Sofia, Bulgaria; 3 Institute of Polymers, Bulgarian Academy of Sciences, Akad. G. Bonchev Str., Bl. 103-A, 1113 Sofia, Bulgaria; s.bozhilova@polymer.bas.bg (S.B.); dchristo@polymer.bas.bg (D.C.); 4 Faculty of Physics, University of Sofia “St. Kliment Ohridski”, 5 James Bourchier Blvd., 1164 Sofia, Bulgaria

**Keywords:** annealing temperature, PVA-*g*-PDMA, polymers, polymer brushes, thin gel films, humidity sensing, optical sensors, colorimetric sensing

## Abstract

Thermal annealing improves the mechanical, structural, and electrical properties of polymer thin films, promoting processes like residual solvents and stress removal, as well as the crystallization and densification of the gel layer. The effects are strongly dependent on the annealing temperature, where optimal temperatures enhance film performance, while excessive thermal exposure may induce negative outcomes like amorphous structural transitions, increased roughness, and defect formation. In this work, thin films of two humidity-sensitive poly(vinyl alcohol) (PVA)-based copolymers with grafted poly(N,N-dimethylacrylamide) (PDMA) chains were investigated. The polymers differ in grafting density and chain length, enabling the assessment of macromolecular architecture’s effects. Spin-coated films with 150–200 nm thickness were annealed at three temperatures: 60 °C, 120 °C, and 180 °C. By using UV-VIS-NIR spectroscopy and the quartz crystal microbalance method, a comprehensive characterization of temperature- and humidity-induced changes in swelling, hysteresis, sensitivity, detection resolution, and water uptake is performed, elucidating the role of the macromolecular architecture on the post-deposition annealing modification of gel film properties and its humidity response. High-performance humidity sensing with a resolution of 0.8% RH is achieved through the optimization of the interplay between the macromolecular architecture and annealing temperature. In addition, the study highlights and explores the potential of these films for optical color-based moisture detection.

## 1. Introduction

Thermal annealing is a widely used strategy for tailoring the morphology, density, and microstructure of polymer thin films, thereby regulating their interaction with moisture and performance in sensor applications. Key influences of temperature annealing include enhanced solvent resistance and stability, morphological and structural changes, modified hygrosensitive properties, and/or improved mechanical and electrical characteristics. Depending on the intended application and sensor configuration, its characteristics are able to be adjusted depending on the desired effect. For example, annealing at or above a critical temperature of the polymer makes the thin films more resistant to the original solvent used during preparation. For lower annealing temperatures, material loss due to rapid dissolution may occur. This improved stability is crucial for multi-layer device fabrication and long-term performance [1,2,3,4]. Similarly, thermal treatment drives the rearrangement of polymer chains, leading to physical changes in the film’s structure, like increased density (annealed films generally become denser, reducing the free volume concentration between polymer chains), crystallization and grain growth (in semi-crystalline polymers, annealing can induce or enhance crystallization, leading to larger, more ordered crystalline regions or grain sizes) [1,4,5,6,7,8], surface alterations (the film surface morphology can evolve from irregular nanocrystals to more defined structures and sizes, affecting surface energy and roughness), etc. Regarding moisture-sensitive polymers, thermal treatment can lead to modified hygrosensitive properties, as the structural and morphological changes directly affect how the polymer interacts with humidity. Swelling and hydration can be tuned. Temperature influences the film’s ability to adsorb water molecules and undergo swelling [9,10]. For example, some poly(vinyl alcohol) derivatives showed swelling exceeding 100%, and proper annealing optimized their humidity-sensing properties [11]. The changes in density and microstructure alter the film’s optical properties, such as the refractive index, absorption coefficient, and transmittance/reflectance, which can be leveraged for optical humidity-sensing applications. Furthermore, annealing can increase film hardness and Young’s modulus by promoting atomic arrangement [4,7]. Polymer brushes consist of long macromolecules, with a rather rigid conformation constrained by steric interactions among the side chains attached to the backbone [12,13,14]. Thanks to their versatile structure, high density of functional segments, and reversible responsiveness to environmental factors, such as pH, temperature, ionic strength, and light [15,16,17], these materials are promising for applications in health sciences [18,19,20], photonics and plasmonics [14,21,22,23,24], adhesion and friction control [25,26], channel flow [27,28], and water harvesting and humidity sensing [29,30]. The measurement of relative humidity plays a pivotal role in various fields [31]. Humidity-responsive materials are promising for applications including environmental monitoring, smart food packaging, wearable humidity sensors, and optical-sensing platforms requiring simple, low-cost, and real-time humidity detection. Their reversible optical response and tunable sensitivity make them attractive candidates for next-generation colorimetric-sensing devices. These polymers may undergo swelling or collapse in response to external factors related to the surrounding media. The physical properties of brush films may also change as a result of melting and glass transitions. Temperature-induced transitions, such as glass–glass transitions or ß-relaxation, may significantly impact wettability, heat capacity, and the refractive index [32]. Recent studies [33,34,35] have demonstrated that polymer brush architectures undergo significant humidity-induced conformational rearrangements, including altered hydration profiles and topology-dependent swelling behavior. These effects arise from changes in interchain hydrogen bonding and the hydrophilic/hydrophobic balance within the brush layer. Principally, both grafting density and chain length govern the thermal relaxation and water adsorption capacity of polymer brushes, as high grafting density and long chains restrict segmental mobility and slow relaxation while reducing water uptake, while lower densities and shorter chains allow easier relaxation and higher water accessibility. In essence, annealing is a critical processing step for tailoring the physical and chemical properties of hygrosensitive polymer thin films to meet specific performance requirements for applications like advanced humidity sensors and multi-layer electronic devices. Despite the progress and the developments in this field, the systematic regulation of humidity sensitivity through annealing of grafted polymer brush films remains insufficiently explored, particularly for double-hydrophilic graft architectures where water uptake, swelling kinetics, and hysteresis are strongly coupled to nanoscale chain organization.

In our previously published research, we have already demonstrated that annealing temperature affects the optical and sensing characteristics of thin films of amphiphilic poly(vinyl alcohol) copolymers, such as poly(vinyl alcohol-*co*-vinyl acetal)s [11] and poly(N,N-dimethyl acrylamide)-poly(ethylene oxide) (PDMA/PEO)—copolymers of different compositions and structures [36]. Extending this investigation to double-hydrophilic brush copolymers is therefore well justified. Moreover, the analysis of temperature-induced effects should be broadened through additional measurements and methodologies, including water uptake determination and the evaluation of swelling–deswelling hysteresis as examples.

In the present paper, we study how different annealing temperatures affect the amounts of absorbed water, optical characteristics, volume dimensions, and sensing properties of hygrosensitive thin films of two poly(vinyl alcohol) (PVA) derivatives comprising grafted poly(N,N-dimethylacrylamide) (PDMA) chains of varied length and graft density. The synthesis, characterization, and general properties of the copolymers have been described in our previous publication [37]. When deposited on the substrate surface, the entangled copolymer chains form gel-like films physically cross-linked through hydrogen bonding and hydrophobic interactions; therefore, thermal annealing represents a viable approach for tuning their sensing properties. The thin films, with a thickness between 150 and 200 nm, were deposited by the spin-coating method. By combining optical characterization with quartz crystal microbalance (QCM) measurements, we evaluated how annealing at 60 °C, 120 °C, and 180 °C modifies the refractive index, density, water uptake, sensitivity, and swelling–deswelling hysteresis across a wide dynamic range. The results demonstrate the potential of these materials as a receptor medium for optical colorimetric humidity sensing and clarify the role of thermal treatment in controlling the humidity response of grafted polymer brush films.

## 2. Results and Discussion

### 2.1. Optical Properties of the Copolymer Thin Gel Films Heated at Different Temperatures

**Figure 1:**
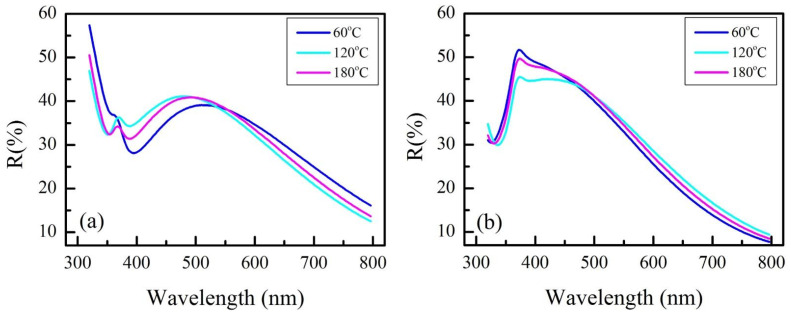
The reflectance spectra of the G1 (**a**) and G2 (**b**) films thermally treated at 60 °C (dark blue line), 120 °C (cyan), and 180 °C (magenta) at room temperature and 50% RH.

Solutions of the two copolymers, each at a concentration of 2%, were used to deposit spin-coated nanosized films on Si wafers and quartz resonators. To ensure that the initial states of the films are comparable, deposition was carried out using a fresh copolymer solution. All samples were deposited from the same solution and at the same time. Annealing took place after deposition at the three chosen temperatures (60 °C, 120 °C, and 180 °C). Firstly, the reflectance spectra R were measured at room temperature and humidity level 55% RH in order to calculate optical constants and thicknesses of the obtained films. The spectra are shown in Figure 1.

**Figure 2:**
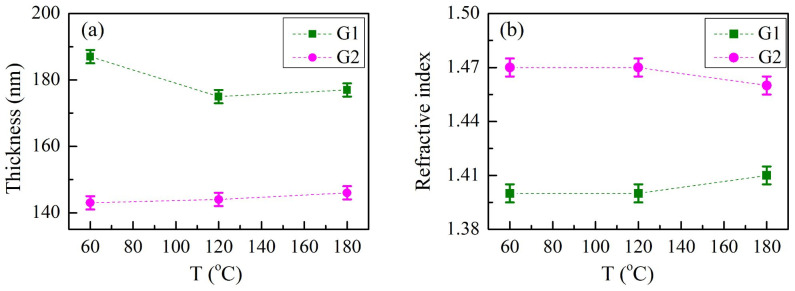
Calculated thickness *d* (**a**) and refractive index *n* (**b**) with the corresponding errors as vertical bars of the G1 (green squares) and G2 (pink circles) films thermally treated at 60 °C, 120 °C, and 180 °C.

The reflectance spectra of the G1 sample exhibited more pronounced temperature-dependent changes compared to the G2 films, indicating that the thermal treatment affected the G1 copolymer more strongly than G2. This was also confirmed by the calculated thicknesses d and refractive indices *n* (Figure 2). Although the two copolymers’ solutions used for film deposition had the same concentration (2%), the resulting thin films had different thicknesses and refractive indices due to the differences in grafting density and length of grafted PDMA side chains. The lower grafting density of G2 suggests that its chains undergo stronger compression during the thin film deposition process, which leads to a smaller thickness of the films (Figure 2a).

This results in increased packing density and manifests in a higher refractive index (Figure 2b). In contrast, the G1 film, whose polymer chains are distributed more densely, undergoes less compression upon deposition and therefore forms a thicker layer. This lowers the packing density and results in a reduced refractive index [37]. Figure 2a shows that annealing the G1 sample at 120 °C resulted in significant shrinkage—the thickness decreases from 187 nm to 175 nm. However, this thickness reduction is not accompanied by change in the refractive index. No further shrinkage was observed for annealing at 180 °C, but a slight increase in *n* was detected.

For the G2 sample, there is no noticeable change in *n* and *d* with annealing, except with a small increase in *n* at 180 °C that was within the experimental error. The different thermal responses for the G1 and G2 samples can be explained by the interplay between chain length and grafting density. For both films, annealing at 120 °C induces chain relaxation and promotes the removal of residual free volume and trapped solvent. The higher grafting density of the G1 polymer allows for limited conformational rearrangements. During relaxation, chains may tilt slightly toward the substrate, collapsing the small gaps between them. This results in a decrease in thickness, while the refractive index remains nearly unchanged, and the overall mass density and chemical composition of the film are preserved. No further change in thickness (*d*) was observed upon annealing at 180 °C, suggesting that the polymer film has reached a geometrically and mechanically stable state. The small increase in *n* from 1.40 to 1.41 observed at 180 °C falls within the uncertainty of the measurement (+/−0.005), indicating no meaningful variation in *n* at this temperature. In contrast to G1, longer, more loosely grafted chains of the G2 film can accommodate thermal relaxation through internal reorganization rather than collective tilt, as was the case with G1. Consequently, no changes in thickness or *n* were observed at 120 °C, while annealing to 180 °C resulted in a slight decrease in *n* from 1.47 to 1.46. However, similarly to the case discussed above with G1, this change is attributed to measurement uncertainty rather than a real change in the material properties. The absence of thickness changes for the G2 sample at both temperatures reflects the fact that these long, loosely grafted chains can accommodate thermal relaxation internally, without macroscopic swelling or contraction.

In summary, the G1 films consist of short, densely grafted polymer chains that are strongly stretched away from the substrate. This leads to a non-equilibrium “upright” brush-like configuration, which relaxes upon annealing at 120 °C through cooperative chain tilting, resulting in a decrease in film thickness. Such orientational relaxation leads to a decrease in film thickness. At the same time, the nearly constant refractive index suggests only minimal variation in the average packing density of the film. In contrast, the chains in G2 are longer, more loosely grafted, and less strongly stretched away from the substrate. Because the average interchain distance is larger, interactions between neighboring chains are weaker, providing the chains with substantially greater conformational freedom already in the as-prepared state. Consequently, the initial brush is closer to its thermodynamically preferred conformation. Upon annealing, relaxation occurs primarily through internal conformational rearrangements, including backbone torsional motions and segmental fluctuations. Importantly, these motions take place mainly within the chain contour rather than through collective reorientation of entire chains. As a result, the long chains are able to accommodate thermal stress internally, and no significant macroscopic changes in either thickness or refractive index are observed.

### 2.2. Optical Humidity Detection

**Figure 3:**
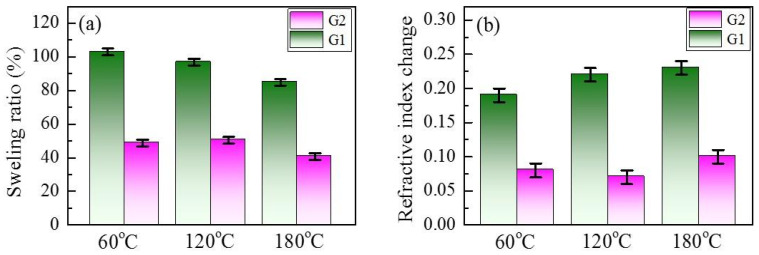
Swelling ratio (**a**) and refractive index change (**b**) with corresponding error bars of the G1 (green bars) and G2 (pink bars) films thermally treated at 60 °C, 120 °C, and 180 °C and exposed from low (5%) to high (95%) relative humidity.

Optical detection allows for rapid, localized, and non-contact moisture assessment, with swelling and hysteresis serving as physicochemical indicators of the quality and dynamics of the polymer film. The swelling ratio of the investigated PVA-*g*-PDMA copolymers is directly related to the swollen film thickness and is strongly influenced by the chain length and grafting density of the side chains. The swelling ratio is a measure of volume change that directly determines the film thickness. As a PVA-*g*-PDMA copolymer absorbs water and its swelling ratio increases, its volume expands, leading to a proportional increase in thickness. The optimal performance is achieved by identifying the annealing temperature that minimizes hysteresis while maximizing color change, sensitivity, and operating range. Samples treated with different temperatures showed different responses to moisture exposure. Figure 3 shows the dependence of humidity-induced swelling and refractive index modulation in the thermal history of the samples.

When temperature of post-deposition annealing increases, sample G1 exhibits a slight gradual deterioration in humidity-induced swelling, decreasing from 103% at 60 °C to 97% at 120 °C and further to 85% at 180 °C. In contrast, sample G2 shows nearly identical swelling ratios at 60 °C and 120 °C (≈50%), followed by a decrease to 41% at 180 °C. The different thermal and humidity-induced swelling behaviors of the two copolymer architectures indicate that in short, densely grafted polymer brushes (G1), thermal annealing induces irreversible densification that suppresses subsequent humidity-induced swelling, even in the absence of measurable changes in dry thickness, as observed at 180 °C. In contrast, in long, loosely grafted polymer brushes (G2), thermal annealing mainly affects internal chain packing and reorganization rather than the macroscopic brush height, resulting in diminished humidity-induced swelling only after annealing at sufficiently high temperatures like 180 °C.

When exposed to high humidity, the gel film swells, leading to an increase in thickness. This reduces the polymer mass per unit thickness, leading to a decrease in the effective refractive index. Consequently, if the swelling ratio decreases with annealing temperature (as in the case of the G1 sample), one would generally expect a corresponding decrease in refractive index modulation, since smaller thickness changes should produce smaller changes in the refractive index. However, the trend observed in Figure 3b is the opposite. For G1 films annealed at 120 °C, where swelling is suppressed compared to 60 °C, a larger refractive index contrast between dry and wet states is observed. This can be explained by water uptake, which occurs alongside volume changes. Water fills the free volume and replaces residual air gaps in the film, increasing the effective refractive index. Thus, two competing processes—volume (thickness) change and water uptake (composition change)—determine the final value of Δ*n*, with their balance controlling the observed refractive index modulation.

To further evaluate the humidity-responsive behavior of the copolymer gels, repeated humidity cycles were performed, confirming the reversible swelling/deswelling behavior of the investigated gel films and their immediate optical response to humidity variation. The cyclic adsorption/desorption behavior was evaluated in order to confirm the reversibility and repeatability of the humidity response. Consecutive humidity cycles (5–95% RH) were performed, demonstrating fully reversible behavior with overlapping adsorption/desorption branches and no observable signal degradation or baseline drift. This behavior was found to be consistent for all investigated samples and remained unchanged throughout the study, confirming the robustness and reproducibility of the sensing response.

**Figure 4:**
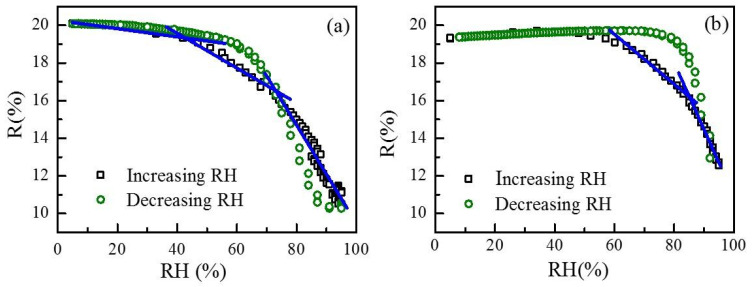
Reflectance versus relative humidity levels of G1 treated at 120 °C (**a**) and G2 treated at 180 °C (**b**) when exposed to humidity increasing from 5% to 95% (black squares) and vice versa—from 95% to 5% RH (green circles). Linear regions are marked with blue lines.

Furthermore, the hysteresis (*H*), sensitivity (*S*), and resolution of the thin films were calculated. The results are summarized in Table 1. Calculations were made from the measured *R*-vs.-*RH* curves in adsorption/desorption regimes, which show the change in reflectance as a function of humidity level (Figure 4).

Figure 4 shows only the results of G1 treated at 120 °C (Figure 4a) and G2 treated at 180 °C (Figure 4b), as these temperatures correspond to the minimum hysteresis for G1 and the highest sensitivity/accuracy for both samples. The measured *R*-vs.-*RH* curves (process of swelling–deswelling) revealed that the thickness change in the copolymer coatings is reversible, which was made as a conclusion from the fact that the end of the desorption curve coincides with the beginning of the adsorption curve. With this in mind, we can confirm that the swelling/deswelling process or thickness change is fully reversible and no additional regeneration of the thin films is necessary. As shown in Figure 4 and according to the values presented in Table 1, the linear regions for the two copolymers are different. The slope of these straight lines shows the sensitivity in a given range and, accordingly, a conclusion can be drawn as to which humidity ranges the thin films can detect the change in the humidity and with what accuracy. For example, G1 samples annealed at 60 °C and 120 °C can be used to measure relative humidity over a wide range (5–95% RH), with different sensitivity and accuracy (see Table 1). In contrast, G1 samples annealed at 180 °C and G2 samples annealed at all investigated post-deposition temperatures are suitable only for measuring *RH* values above 60%. Among them, the application of G2-180 in the 65–95% RH range provides the highest accuracy.

Regarding the degree of hysteresis, it is seen that, for G1 sample, the percentage of *H* is lowest at an annealing temperature of 120 °C, but it is still very close to the one at 60 °C. As the temperature increases to 180 °C, the effect worsens—*H* changes from 7–8% to 11%. Because hysteresis originates from faster desorption than sorption, it is primarily governed by delayed hydration or slow polymer relaxation during the sorption process. The as-prepared G1 film (dried at 60 °C) exists in a non-equilibrium state, likely containing non-equilibrium chain conformations, heterogeneous free volume, and constrained diffusion pathways. However, water sorption requires water molecules to diffuse into the film, open intermolecular spaces, and induce local chain rearrangements. Because G1 features a brush-like structure with densely grafted PDMA side chains, these structural adjustments are kinetically hindered. Consequently, sorption proceeds more slowly than desorption, leading to high hysteresis. Annealing at 120 °C—a temperature above the glass transition temperatures (T_g_) of both the PVA (T_g_ ≈ 80 °C) and PDMA (T_g_ ≈ 100 °C) components—facilitates overall structural relaxation. As a result, the polymer film adapts more rapidly during water uptake, which reduces hysteresis.

When the G1 film is annealed at 180 °C, the grafted PDMA chains enter a fully mobile liquid-like state, as this temperature is well above its T_g_ (≈100 °C) and far below the onset of thermal degradation (≈300 °C). At this elevated temperature, the short PDMA grafted chains exhibit high segmental mobility and adopt amorphous, random-coil conformations, which results in a more tightly packed structure upon cooling. Under these conditions, hydration becomes kinetically hindered once again because water uptake now requires the cooperative opening of a denser matrix. At the same time, desorption remains relatively rapid because the contraction of the brush structure is energetically favorable, ultimately leading to an increase in hysteresis.

The *H* values for G2 samples treated at the three different temperatures do not change, remaining at the same unchanged value of 6%. This is assigned to the more loosely grafted copolymer structure and the greater conformational freedom of the long PDMA side chains that allow water uptake to be accommodated through localized intrachain rearrangements, without requiring collective structural relaxation. Consequently, the sorption/desorption kinetics remain largely unaffected by annealing, and hysteresis remains nearly constant.

**Figure 5:**
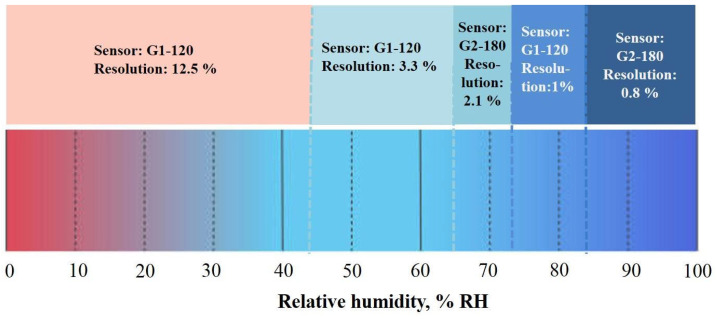
Selected polymer films for relative humidity sensing over the 0–100% RH range with the highest resolution and the smallest hysteresis.

Figure 5 illustrates the selection of the most suitable copolymer gel films for relative humidity measurements over the full range (0–100% RH), achieving both the highest resolution and the lowest hysteresis. The presented results demonstrate that the humidity-responsive behavior can be tuned through the copolymer structure and annealing conditions, leading to optimal performance of different films in different *RH* regions. In particular, the combination of G1 annealed at 120 °C and G2 annealed at 180 °C enables effective humidity detection across the entire investigated humidity range, as summarized below:

✔ 0–42% RH: Resolution 12.5%, sensor G1-120
✔ 42–64% RH: Resolution 3.3%, sensor G1-120
✔ 64–74% RH: Resolution 2.1%, sensor G2-180
✔ 74–84% RH: Resolution 1.0%, sensor G1-120
✔ 84–100% RH: Resolution 0.8%, sensor G2-180

In the low humidity region (0–42% RH), the measurement accuracy is relatively poor (≈12.5%). However, such low humidity levels are uncommon under typical atmospheric conditions, and therefore this limitation does not significantly affect the practical applicability of the sensor. All these changes also lead to a change in the color of the thin film samples. The color variation in polymer film upon humidity-induced swelling is directly related to changes in its optical thickness (product of *n* and *d*). When exposed to moisture, hydrophilic polymer films absorb water molecules, leading to volumetric swelling and an increase in physical thickness *d*. Simultaneously, the effective refractive index decreases, driven by a reduction in polymer volume density upon swelling and by a drop of the polymer’s optical polarizability due to “dilution” with lower refractive index water.

Although the thickness increase and refractive index decrease act in opposite directions, the swelling-induced thickness change typically dominates, resulting in a red shift in the reflectance interference maxima as humidity increases, i.e., a shift toward a longer wavelength is observed. Consequently, the humidity-dependent color variation serves as a direct optical indicator of the film’s swelling behavior and refractive index modulation. This mechanism underpins the operation of colorimetric and optical humidity sensors based on thin polymer films, enabling label-free, real-time, and visually detectable moisture sensing. Considering that, for G1 and G2, the humidity-induced change in the refractive index (Figure 3b) is smaller as compared to thickness changes (Figure 3a), we can conclude that thickness change in both cases prevails and the color change is mostly caused by swelling.

**Figure 6:**
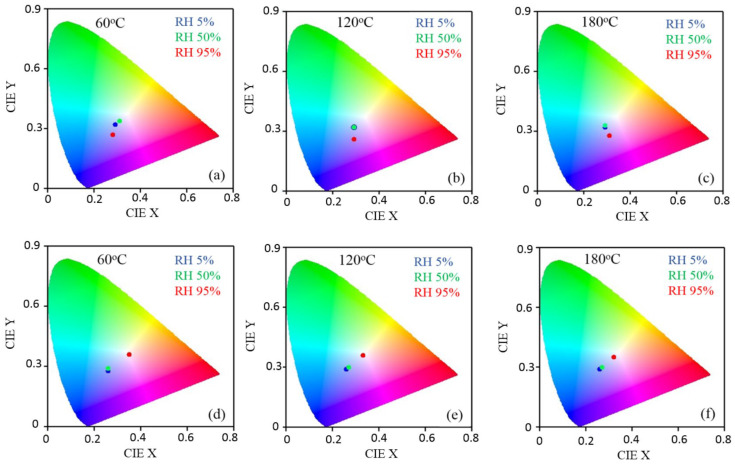
Color coordinates of G1 (**a**–**c**) and G2 (**d**–**f**) thin film samples when exposed to 5% (blue dots), 50% (green dots), and 95% (red dots) relative humidity.

As can be seen from the graphs with the color coordinates (Figure 6) for all samples, the greatest color change is observed at high humidity (Figure 6, red dots). The points at 95% RH are at the furthest distance from the points indicating the color at humidity 5% and 55% RH (Figure 6, blue and green dots) and in a completely different area/color of the color diagram. None of these overlap and are not close to the others. At low and medium humidity—5% and 55% RH—the color change is not very distinguished, but there is still a difference, which is most clearly expressed with the sample G1 heated at 60 °C. Color coordinates for the G1 sample heated at 60 °C are sufficiently spaced apart and, even at the low humidity of 5% RH, the sample exhibits a different color, while the color coordinates at 5% and 55% RH do not overlap.

The results for sample G2 indicate that the temperature does not affect the color coordinates, and for all three annealing temperatures, the points are almost identical. The same trend of shift is observed in the order of blue point (5%), green point (50%), and red (95%). It can be seen from Figure 6a that sample G1 heated to 60 °C demonstrates the most clearly separated color coordinates at the three humidity levels.

### 2.3. Hydration of the Materials

After each annealing step, hydration was continuously monitored using the QCM (quartz crystal microbalance) device. Before use, the new resonators were washed with water and ethanol. In a standard experiment, a homemade bubbler system was used to characterize the bare resonator, using a QCM device equipped with a temperature-controlled module under dry argon at 5% RH. The resonator was then removed from the QCM, and a thin layer was spin-coated on the surface using the same method to produce polymer layers on Si substrates, rotating at 4000 rpm for 60 s. The spin-coating technique was employed in order to obtain thin evenly distributed films across the resonator surface. A homemade bubbler system connected to the QCM cell was utilized to assess the sorption and desorption of water vapor by the copolymer gel films after the resonator was inserted into the QCM module. The films were continuously exposed to increasing/decreasing RH values in the range [5–95%] at a constant temperature T = 20 °C. The frequency behavior associated with water vapor sorption/desorption kinetics observed for polymer layers G1 and G2 deposited on a quartz resonator and heated at 120 °C and 180 °C was similar to that for the annealed at 60 °C samples, as previously demonstrated in [37].

A reversible shift in the measured frequency is observed when RH changes. Since the four measured harmonics are closely spaced for both polymers—a behavior characteristic for samples close to the Sauerbrey regime, suggesting that the thin layers are slightly viscoelastic [38]—for the adsorbed mass calculation, we applied the model for a single viscoelastic film in air [38,39,40]:

(1) $$\frac{{\Delta f}_{N}}{N}=\;-\frac{2m_{f}f_{0}^{2}}{Z_{q}}\left(1+\frac{Z_{q}^{2}m_{f}^{2}\pi^{2}N^{2}}{3Z_{f}^{2}m_{q}^{2}}\right),$$

where *Z_f_* is the acoustic impedance of the film (kg·m^−2^·s^−1^), and *m_q_* is the areal mass density of quartz (kg·m^−2^).

**Figure 7:**
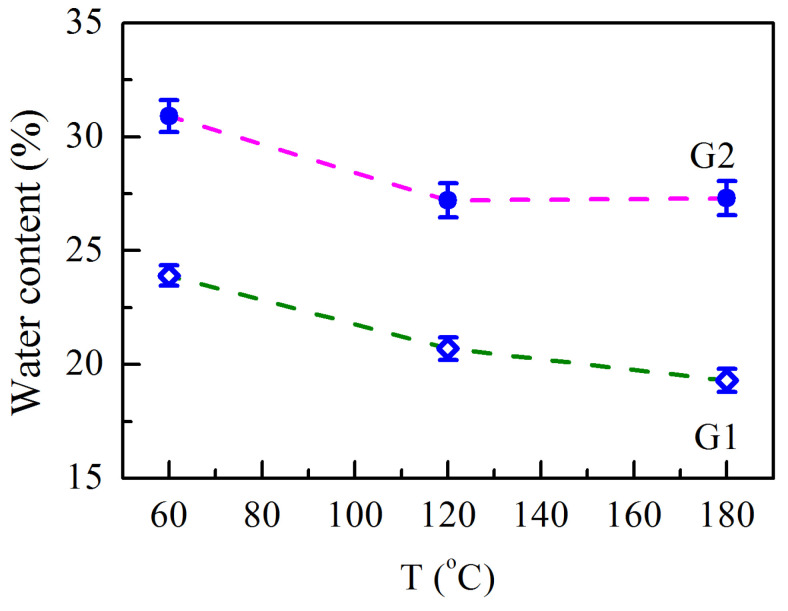
Water content at 95% RH of G1 and G2 samples, with experimental uncertainties as error bars as a function of post-deposition annealing temperature.

The model was selected because, in the simplest case where there are no differences between the normalized frequency shifts in the overtones, it simplifies to the Sauerbrey model, whereas if differences appear in more complex viscoelastic cases, it yields a more accurate mass determination [38]. To evaluate the moisture accumulated in the polymer matrix, measurements were also performed on a bare silica resonator under identical conditions, following the same experimental procedure used for the polymer coated resonator. The water content stored into the copolymer matrix was calculated (Equation (8)) and the results are presented as a function of post-deposition annealing temperature in Figure 7.

For both copolymer gels, the annealing led to a reduction in water sorption capacity. Depending on the macromolecular structure, polymeric materials frequently have free volume that may be filled with molecules. Annealing in air is frequently non-equilibrium and the heating rate, hold time, and cooling path determine the final structure. Free space within the brushes significantly changes the thermodynamics of vapor sorption and brush response, including brush dynamics and vapor sorption kinetics, as multiple data demonstrate [17]. Dry thermal annealing may change segmental mobility, driving reorganization and leading to brushes’ internal packing and steric hindrance for water molecules.

In our case, the annealing effect was most significant at 120 °C, as the next annealing step had a minimal impact on the sorption capacity for G1 film and almost no effect in the case of G2 films (Figure 7). Moreover, for each annealing step, as the polymer with more closely grafted brushes, G1 was estimated to adsorb less water conversely to the more loosely grafted, but longer PDMA brushes in case of G2 led to higher moisture being regained and stored in the polymer matrix. However, the higher film density (see Figure 2b) restricted the film’s ability to swell considerably, leading to smaller changes in thickness compared to G1 films when exposed to high relative humidity. The higher absorption capacity of the G2 films could be attributed to the higher number of polymer chains per unit volume and the resulting increased number of active absorption sites.

## 3. Conclusions

Post-deposition annealing affects densely (G1) and loosely grafted (G2) PVA-*g*-PDMA gel films in distinctly different ways. For G1, annealing at 120 °C leads to a clear reduction in film thickness, with no further changes observed after annealing at 180 °C. In contrast, G2 shows no significant changes in thickness or refractive index upon annealing. This behavior is attributed to differences in chain architecture, where the short, densely grafted chains of G1 relax through collective tilting toward the substrate, leading to film densification and thickness reduction, while the longer, more loosely grafted chains of G2 accommodate thermal relaxation through internal reorganization, without macroscopic structural changes.

At high relative humidity (95% RH), G1 shows a gradual decrease in swelling with increasing annealing temperature, while G2 displays a noticeable reduction in swelling only after annealing at 180 °C. Annealing at 120 °C suppresses water uptake in both G1 and G2, while further annealing at 180 °C has only a minor effect. Overall, water uptake is stronger in G2 because it is more densely packed, thus having more absorption sites within the film volume. These results demonstrate that short, densely grafted polymer brushes undergo irreversible densification upon annealing, which suppresses humidity-induced swelling. In contrast, in long and loosely grafted polymer brushes, thermal annealing primarily modifies internal chain packing and hydration thermodynamics rather than the macroscopic dimensions.

Based on these findings, a combination of two gel films—G1 annealed at 120 °C and G2 annealed at 180 °C—is proposed for high-resolution humidity sensing over a wide dynamic range, achieving a resolution of approximately 0.8% RH under high humidity conditions. Furthermore, the feasibility of color-based detection is demonstrated, highlighting the potential of these materials for simple and user-friendly humidity sensors.

## 4. Materials and Methods

### 4.1. Synthesis of Hygrosensitive PVA Copolymers with Grafted PDMA Chains

**Figure 8:**
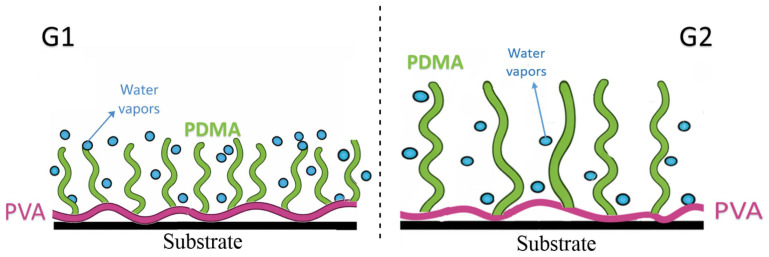
Schematic illustration (non-scaled drawing) of the structure of copolymers G1 and G2 deposited on the substrate surface.

The double-hydrophilic brush copolymers investigated in this work were synthesized by grafting N,N-dimethylacrylamide (DMA) on PVA through redox polymerization in aqueous media, using deionized water as a solvent and ammonium cerium (IV) nitrate as an initiator. The synthesis and characterization procedures are described in detail in our previous publication [37]. Briefly, two copolymers (named G1 and G2) of similar copolymer compositions, but differing in grafting density and length of grafted PDMA side chains, were prepared by using different monomer-to-PVA-to-initiator molar ratios of 1.0:0.23:0.023 and 1.0:0.23:0.012. The resulting copolymers differed only in macromolecular architecture: G1 was more densely grafted with shorter PDMA chains, whereas G2 was more loosely grafted with longer PDMA chains (Figure 8). Chemical composition and structure of the copolymers were evaluated by NMR and FTIR spectroscopy using TGA and DLS measurements [37].

### 4.2. Thin Film Deposition Conditions

The thin films were deposited using a spin-coating method (WS-650-23 B, Laurel, MS, USA). Silicon wafers (for spectrophotometric measurements) and 5 MHz quartz resonators with SiO_2_-coated Au electrodes, which were used in acoustic measurements via quartz crystal microbalance method with an open QCM NEXT device. The resonators and the QCM device were purchased from Novaetech, Pompei, Italy. Copolymer solutions of G1 and G2 were prepared at a concentration of 2 wt% in a water/methanol mixture (20:80 *v*/*v*). The same deposition conditions were applied to both substrates—the copolymer solution was dropped on a pre-cleaned substrate and then rotated at speed of 4000 rpm for 60 s. Due to different area sizes of the substrates, 200 μL of the copolymers were dropped on Si wafers and 100 μL on resonators. Subsequent thermal treatment in air was performed at three annealing temperatures (60, 120, and 180 °C) for 30 min at a rate of 10 °C per minute.

### 4.3. Characterization of the Thin Films

Reflectance spectra (R) of the samples deposited on Si substrates were taken in the range 320–900 nm with a spectrophotometer (UV-VIS-NIR, Cary 5E, Varian, Mulgrave, Australia). The collected spectra were used to calculate the optical constants of the thin polymer films (refractive index *n* and absorption coefficient *k*) and thickness of the films (d), using the previously developed two-stage nonlinear curve fitting method in our working group [41]. All measurements were repeated at least three times under identical experimental conditions, and the presented values represent the average values obtained from these repeated measurements. The error bars shown in the figures correspond to the standard deviation of the measured/calculated parameters. Experimental uncertainties originate mainly from reflectance measurements, nonlinear fitting procedures, humidity stabilization, and thickness/refractive index calculations. The errors in thickness and the refractive index are ±2 nm and ±0.005, respectively. The error bars shown in Figure 2 represent the standard deviation calculated from at least three repeated measurements, and the uncertainty originated from the measurement error of reflectance.

### 4.4. Sensing Properties

**Figure 9:**
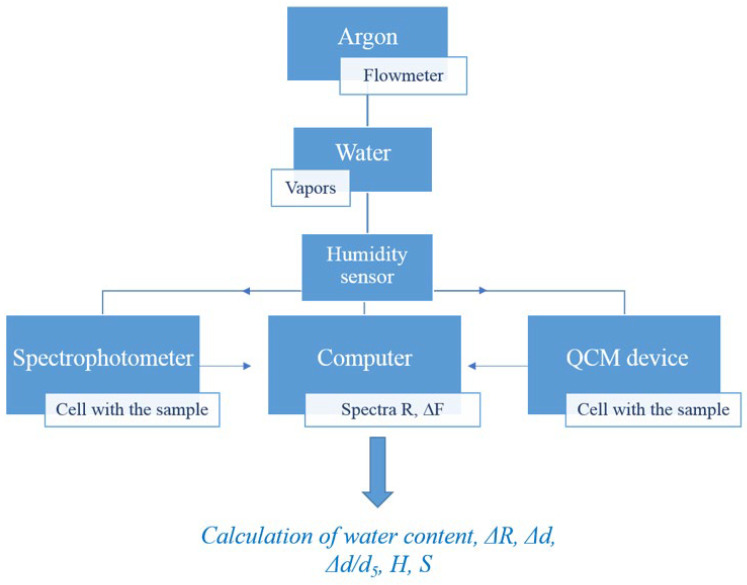
Scheme of the measurement process and evaluation of humidity properties of thin films.

For the evaluation of the sensing properties of the films by means of spectrophotometry via the quartz microbalance method, the same setup for humidity control was used. The setup scheme is shown in Figure 9.

Briefly, water vapor is generated using a homemade bubbler system, and different relative humidity levels are produced by argon flow through distilled water maintained at 60 °C and directed into the sample cell. Samples deposited on Si wafers are placed in a quartz cell positioned in the spectrophotometer, allowing the spectra to be measured while humidity level is changing to the desired levels. The same procedure was applied to copolymer gel films deposited on quartz resonators.

#### 4.4.1. Optical-Sensing Properties

Firstly, for the estimation of the changes in the polymer films via spectrophotometric method, the reflectance spectra *R* at different humidity levels are taken and the wavelength λ_max_ of the highest humidity response Δ*R*_max_ is established. For each sample, determination of the λ_max_ was conducted. The refractive index *n* and absorption coefficient *k*, along with the thickness d of the film and their changes Δ*n* and ∆*d*, were calculated by using the measured reflectance spectra in the range 320–800 nm at 5% and 95% RH:

Δ*d* = *d*_95%_ − *d*_5%_, (2a)

Δ*n* = *n*_5%_ − *n*_95%_, (2b)

where *d*_95%_, *n*_95%_, *d*_5%_, and *n*_5%_ are the thicknesses and refractive indices of the films exposed at the highest humidity level (RH = 95%) and the lowest one (RH = 5%).

The swelling ratio *SR* and thickness change are maximized when the polymer network has a balance between sufficient main chain length/flexibility and long hydrophilic side chains, along with a moderate cross-linking or grafting density that allows for expansion without causing a highly compact impenetrable structure. The interplay between these factors determines the final equilibrium state of the swollen polymer. In order to evaluate the swelling ratio *SR* of thin polymer films when exposed to different humidity levels, the thickness values *d*_95%_ and *d*_5%_ were used in the following formula:

(3) $$SR\;=\;\frac{d_{95\%}-d_{5\%}\;}{d_{5\%}}=\frac{∆d\;}{d_{5\%}},$$

where *d*_95%_ and *d*_5%_ are thicknesses of the film when exposed to 95% and 5% RH, respectively. Recording of *R*-values while humidity levels are changing from 5% RH to 95% RH and vice versa were made at the already chosen λ_max_ in order to determine the hysteresis value *H*. Hysteresis is the difference in the sensor’s output when measuring the same humidity level during an increasing humidity cycle versus a decreasing one. This lag is caused by the sensing material’s memory effect, where the interaction between the sensing material and water molecules is not perfectly reversible, leading to different responses depending on its recent history. So unwanted hysteresis may occasionally arise when relative humidity transitions from low to high or vice versa. Ideally, for our copolymer sensitive material, we aimed at maximal detection sensitivity with minimal percentage of hysteresis. The value of *H* in our case was determined by Equation (4) as follows:

(4) $$H\left(\%\right)=\frac{\frac{1}{Q}\sum_{i=1}^{Q}\left|R_{up}-R_{down}\right|}{∆R}100,$$

where *R*_up_ and *R*_down_ are reflectance values measured for increasing and decreasing humidity, respectively; Δ*R* is the reflectance change in the humidity range 5–95% RH; and Q is the number of points over which the summation is carried out.

Multiple criteria were used to evaluate the optical-sensing properties and quantify the samples under investigation along with the hysteresis. The sensitivity *S* and accuracy Δ*RH* of the films have also been calculated by following equations:

(5) $$S=\frac{∆R\;}{{RH}_{2}-{RH}_{1}},$$

where Δ*R* (%) represents the variation in the reflectance of the thin film when humidity levels transition from *RH*_1_ to *RH*_2_. Then, by using the sensitivity value *S*, the detection accuracy/resolution (Δ*RH*) can be estimated due to its dependence on the sensitivity and measurement accuracy in the signal:

(6) $$\Delta RH\;=\;\frac{errR\;(\%)}{S\;(\%)},$$

where the experimental error (accuracy) of reflectance *R* is *errR* = 0.3%; while *S* is the sensitivity already calculated via Equation (5).

#### 4.4.2. Quartz Crystal Microbalance Method

Factors contributing to hysteresis can be different, but these are mostly related to material properties; the kinetics of water molecule adsorption (sticking) and desorption (releasing), which are not always identical; and even water clusters that might form within the polymer, which can cause deformation and influence the sensor’s material performance. In order to examine the hydration properties of the material and the impact of thermal annealing on the humidity-sensing properties, as well as to better understand the processes that take place when polymers are exposed to different levels of humidity, a quartz crystal microbalance method was used. The study [42], performed by Brochard-Wyart and de Gennes, is a pioneering work on the swelling of polymer brushes by vapor, presenting a theoretical analysis of the capillary rise in a liquid along a brush-covered surface.

Brush-modified QCMs have been employed for various polymer−solvent systems, exhibiting diverse response kinetics and reversibility [17]. The shift in resonance frequency of a piezoelectric device as its mass changes with the adsorption of an analyte is commonly used in gravimetric-sensing techniques when calculating analyte concentration.

Assuming that the material adsorbed on the resonator’s active area is uniformly distributed and rigid, and that its mass is small relative to the mass of the quartz crystal, the Sauerbrey equation can be employed. It expresses a linear relationship between accumulated mass and the induced frequency shift [43]:

(7) $$\frac{{\Delta f}_{N}}{N}=\;-\frac{2m_{f}f_{0}^{2}}{Z_{q}},$$

In this equation, *f*_0_ is the fundamental resonance frequency of the quartz crystal (approximately 5 MHz for the used resonators; *Z_q_* is acoustic impedance of quartz (8.8 × 10^6^ kg·m^−2^·s^−1^); *N* is the overtone number; Δ*f_N_*/*N* is the frequency shift in the Nth overtone normalized to *N*; and m_f_ is the areal mass (kg·m^−2^). The equation allows the calculation of the mass of both the adsorbed layer and the analyte molecules. Under the Sauerbrey approximation, the normalized frequency shifts from each overtone should be equal, and the adsorbed mass can be obtained directly from (Equation (6)) by taking the mean of all values obtained from Δ*f_N_*/*N*.

However, in some cases, due to rheological and/or surface properties, the deposits on the quartz resonator film leads to deviations from the Sauerbrey regime—Δ*f_N_*/*N* varies across the overtones depending on the overtone number. In such cases, alternative methods for data analysis are applied [38,39,40,44,45].

According to the relationship (8), the accumulated water mass *m_water_* and the water content in the film can be calculated by using the total film hydrated mass *m*_95%_ (the mass at humidity level RH = 95%) and its dry mass *m*_5%_ (the mass measured at RH = 5%) [44]:

(8) $$water\;content\;\left(\%\right)=\frac{\;m_{95\%}-m_{5\%}\;}{m_{95\%}}100\;=\;\frac{m_{water}}{m_{95\%}}100.$$

## Figures and Tables

**Table 1:** Percentage of hysteresis (H), dynamic range, sensitivity (S), and accuracy for studied samples G1 and G2 thermally treated at 60 °C, 120 °C, and 180 °C.

Sample	Hysteresis *H* (%)	Dynamic Range (% RH)	Sensitivity *S* (%/% RH)	Accuracy/Resolution (% RH)
G1-60	8	5–95	0.01 (5–50% RH)	30 (5–50% RH)
			0.07 (51–80% RH)	4.3 (51–80% RH)
			0.24 (81–95% RH)	1.25 (81–95% RH)
G1-120	6.5	5–95	0.024 (5–42% RH)	12.5 (5–42% RH)
			0.09 (43–73% RH)	3.3 (43–73% RH)
			0.30 (74–95% RH)	1.0 (74–95% RH)
G1-180	11	60–95	0.07 (60–84% RH)	4.3 (60–84% RH)
			0.34 (85–95% RH)	0.9 (85–95% RH)
G2-60	6	65–95	0.11 (65–85% RH)	2.7 (65–85% RH)
			0.35 (86–95% RH)	0.86 (86–95% RH)
G2-120	5.9	67–95	0.10 (67–85% RH)	3.0 (67–85% RH)
			0.36 (86–95% RH)	0.83 (86–95% RH)
G2-180	6	64–95	0.145 (64–84% RH)	2.1 (64–84% RH)
			0.36 (85–95% RH)	0.83 (85–95% RH)

## Data Availability

The original contributions presented in this study are included in the article. Further inquiries can be directed to the corresponding authors.
